# Supplementary material for: Distinct Hormone Signalling-Modulation Activities Characterize Two Maize Endosperm-Specific Type-A Response Regulators
Source: Plants (Basel). 2022 Jul 30;11(15):1992. doi: 10.3390/plants11151992 (PMC9370639; doi:10.3390/plants11151992)
Supplement: Supplementary file 1 [file plants-11-01992-s001.zip › Suppl Figure 1.pdf]

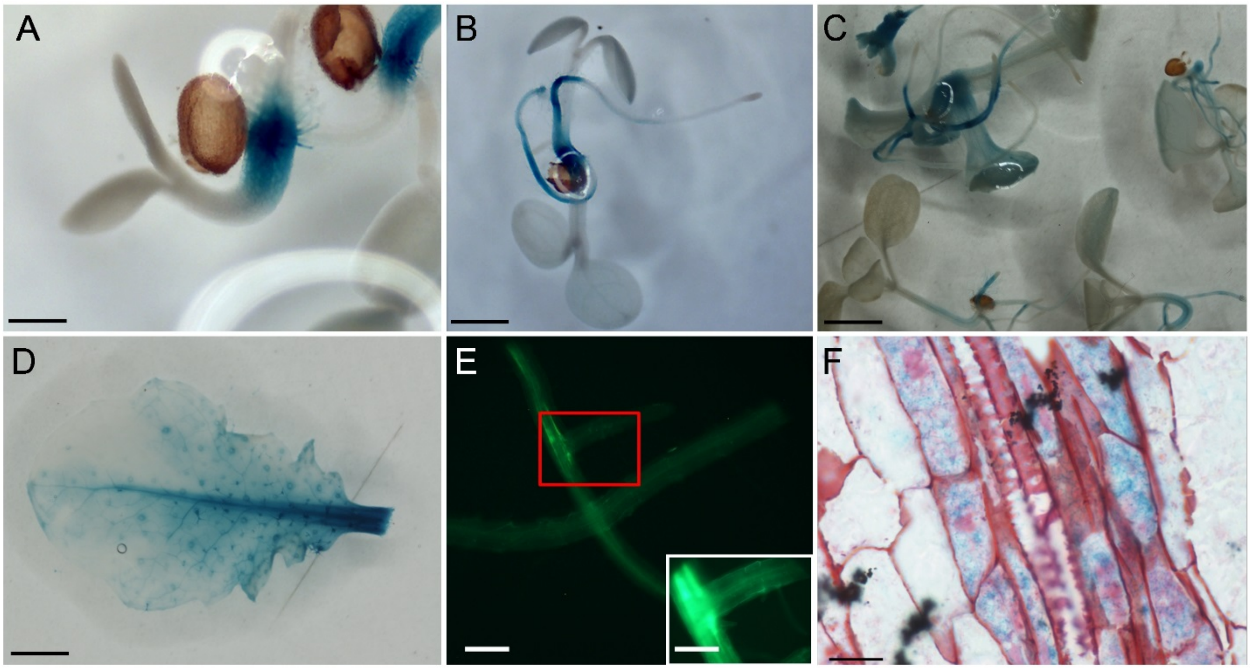

Supplementary Figure S1. The ZmTCRR2promoter labels the vasculature in developing *Arabidopsis*. The panels show plantlets of one representative ZmTCRR2:GFP:GUS line at different developmental stages after germination on MS medium (A-C) or adult leaves (D). A-C, at these initial stages, the signal concentrated at the root and basal hypocotyl and was visible in 1-2 hours after immersion in GUS staining solution; D, rosette leaf of a 3-week-old plant. The signal radiated from the central vasculature and diffused in the mesoderm. E and its inset show the area at the base of a developing secondary root under UV, revealing a more intense GFP fluorescence in two cell files at the base of the growing secondary root. Panel F is a high magnification image of a GUS stained root, displaying the signal in the pericycle cells adjoining to the vasculature. Scale bars represent 1 mm in A-C, 10 mm in E, 200 µm in the inset in E and 20 µm in F.
