# Supplementary material for: Distinct Hormone Signalling-Modulation Activities Characterize Two Maize Endosperm-Specific Type-A Response Regulators
Source: Plants (Basel). 2022 Jul 30;11(15):1992. doi: 10.3390/plants11151992 (PMC9370639; doi:10.3390/plants11151992)
Supplement: Supplementary file 1 [file plants-11-01992-s001.zip › Suppl Figure 2.pdf]

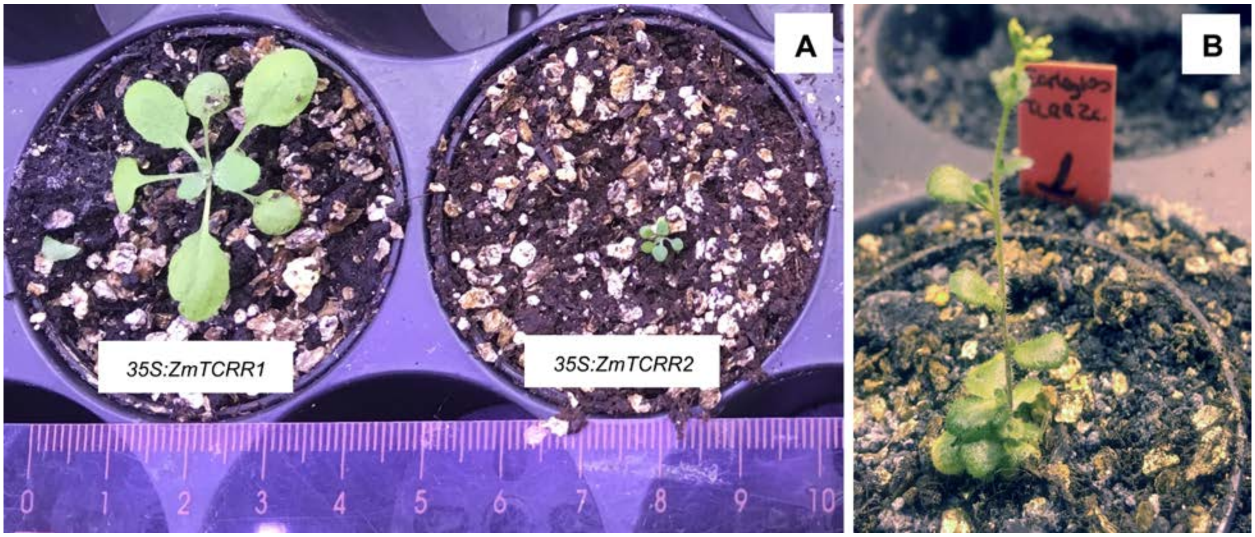

Supplementary Figure S2. *35S:ZmTCRR2* transgenics showed arrested growth and delayed flowering. Panel A shows two transgenic events overexpressing ZmTCRR1 or 2 in soil, 21 days after germination. Note that the plant overexpressing ZmTCRR1 starts blooming, much as a wild-type plant would do. The rule is in cm. Panel B, a transgenic plant overexpressing ZmTCRR2 starts flowering 90 days after germination.
