# Supplementary material for: Distinct Hormone Signalling-Modulation Activities Characterize Two Maize Endosperm-Specific Type-A Response Regulators
Source: Plants (Basel). 2022 Jul 30;11(15):1992. doi: 10.3390/plants11151992 (PMC9370639; doi:10.3390/plants11151992)
Supplement: Supplementary file 1 [file plants-11-01992-s001.zip › Suppl Figure 3.pdf]

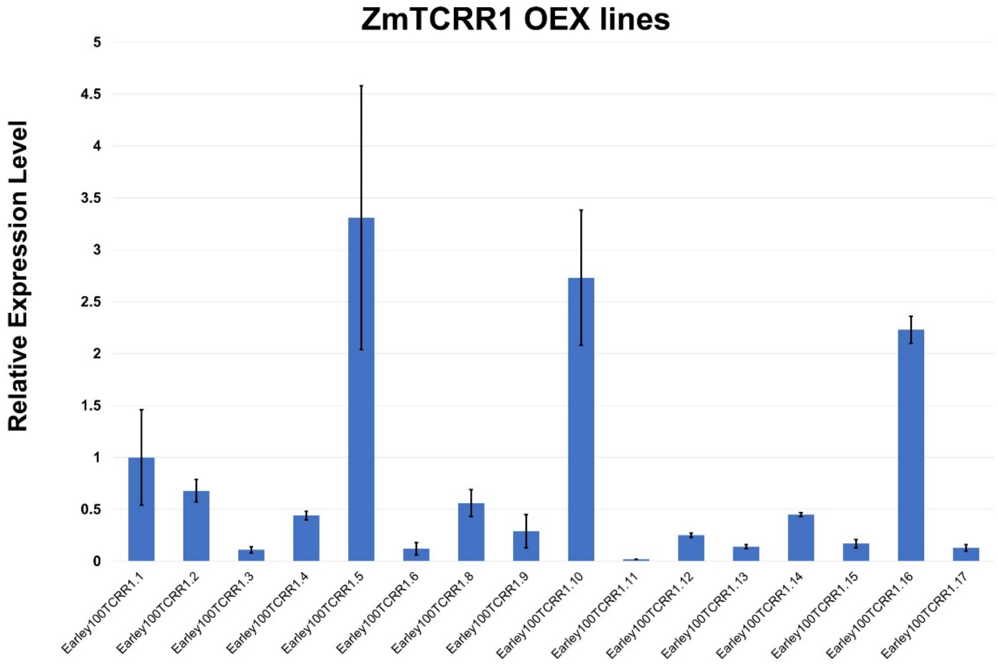

Supplementary Figure S3. *ZmTCRR1* expression analyses in 17 Arabidopsis transgenic lines. RNA extracted from rosette leaves of 15 days old plants was converted into cDNA and analysed for *ZmTCRR1* expression with specific primers, using the ΔΔCT method. A primer pair amplifying the actin genes was used as a normalizer.
