# Supplementary material for: Distinct Hormone Signalling-Modulation Activities Characterize Two Maize Endosperm-Specific Type-A Response Regulators
Source: Plants (Basel). 2022 Jul 30;11(15):1992. doi: 10.3390/plants11151992 (PMC9370639; doi:10.3390/plants11151992)
Supplement: Supplementary file 1 [file plants-11-01992-s001.zip › Suppl Figure 4.pdf]

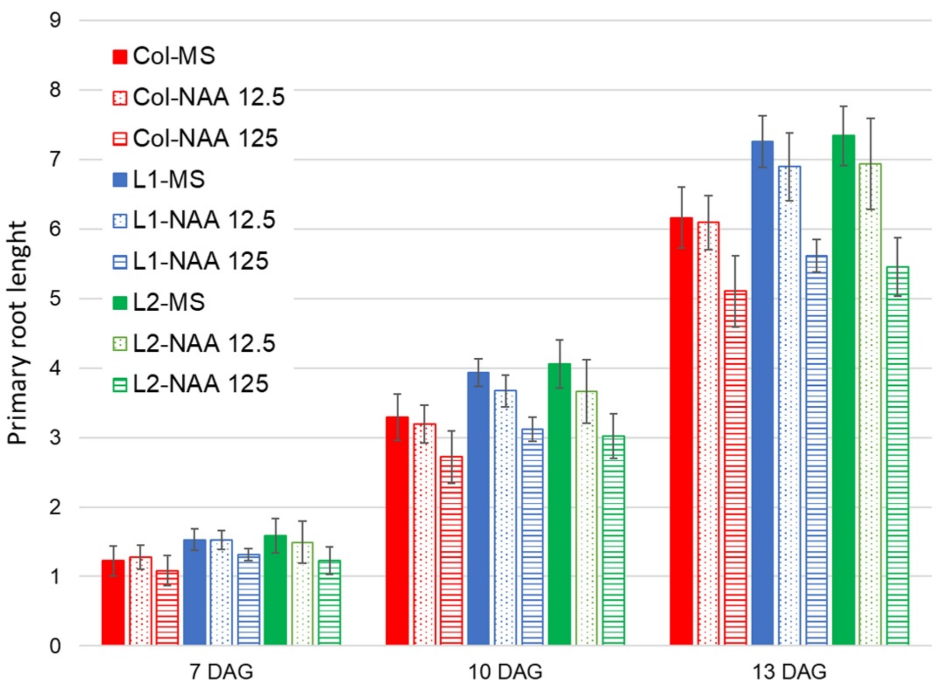

Supplementary Figure S4. ZmTCRR1 interferes with auxin signalling. Wild type (Col) and two independent homozygous transgenic lines (L1, L2) overexpressing ZmTCRR1 were cultured in vertical plates with MS media containing no-hormone, 12.5 nM NAA or 125 nM NAA. The graph shows the average primary root length of each genotype in each culture media after 7, 10, and 13 days. The error bars represent the standard deviation. The transgenic lines show nearly identical behavior and a consistently faster primary root growth. As shown in Figure 5, after 10 days of culture, the growth of ZmTCRR1 plants in the presence of 125 nM NAA is statistically indistinguishable from that of Col in the absence of hormones. Data presented above indicate that the treatment with 12.5 nM NAA produces an intermedia effect in the transgenic lines. However, in most cases, the statistical analyses do not discriminate these samples from untreated or 125 nM NAA treated samples (see statistic calculations in Figure S5).
