# Supplementary material for: Distinct Hormone Signalling-Modulation Activities Characterize Two Maize Endosperm-Specific Type-A Response Regulators
Source: Plants (Basel). 2022 Jul 30;11(15):1992. doi: 10.3390/plants11151992 (PMC9370639; doi:10.3390/plants11151992)
Supplement: Supplementary file 1 [file plants-11-01992-s001.zip › Suppl Table 1.pdf]

Supplementary Table S1. Statistical analyses of the primary root length of plants grown in vertical plates in the presence or absence of BA. For each time point statistically homogeneous groups are defined according to the pairwise comparisons using Bonferroni's test with p≤0.05.

Multiple Range Tests for Primary-root length by Genotype-Treatment. Days 7+0

Method: 95,0 percent Bonferroni

Level	*Count*	*Mean*	*Homogeneous Groups*
**TCRR2-MS**	17	0,755699	X
**TCRR1-MS**	28	1,0486	XX
**TCRR2-5µM BA**	20	1,07036	XXX
**TCRR1-5µM BA**	28	1,10446	XX
**WT-MS**	28	1,36085	X
**WT-5µM BA**	28	1,37773	X

Contrast	*Sig.*	*Difference*	*+/- Limits*
**TCRR1-5µM BA - TCRR1-MS**		0,0558651	0,286529
**TCRR1-5µM BA - TCRR2-5µM BA**		0,0341049	0,313877
**TCRR1-5µM BA - TCRR2-MS**	*	0,348762	0,329637
**TCRR1-5µM BA - WT-5µM BA**		-0,273274	0,286529
**TCRR1-5µM BA - WT-MS**		-0,256385	0,286529
**TCRR1-MS - TCRR2-5µM BA**		-0,0217603	0,313877
**TCRR1-MS - TCRR2-MS**		0,292897	0,329637
**TCRR1-MS - WT-5µM BA**	*	-0,329139	0,286529
**TCRR1-MS - WT-MS**	*	-0,31225	0,286529
**TCRR2-5µM BA - TCRR2-MS**		0,314658	0,353667
**TCRR2-5µM BA - WT-5µM BA**		-0,307379	0,313877
**TCRR2-5µM BA - WT-MS**		-0,29049	0,313877
**TCRR2-MS - WT-5µM BA**	*	-0,622036	0,329637
**TCRR2-MS - WT-MS**	*	-0,605147	0,329637
**WT-5µM BA - WT-MS**		0,0168889	0,286529

* denotes a statistically significant difference.

Multiple Range Tests for Primary-root length by Genotype-Treatment. Days 7+3

Method: 95,0 percent Bonferroni

Level	*Count*	*Mean*	*Homogeneous Groups*
**TCRR2-MS**	17	1,19835	X
**TCRR1-5µM BA**	28	1,74225	X
**WT-5µM BA**	28	1,75173	X
**TCRR2-5µM BA**	20	2,89076	X
**WT-MS**	28	3,47087	X
**TCRR1-MS**	28	3,51996	X

Contrast	*Sig.*	*Difference*	*+/- Limits*
**TCRR1-5µM BA - TCRR1-MS**	*	-1,77771	0,773302
**TCRR1-5µM BA - TCRR2-5µM BA**	*	-1,14851	0,84711
**TCRR1-5µM BA - TCRR2-MS**		0,543893	0,889643
**TCRR1-5µM BA - WT-5µM BA**		-0,00948557	0,773302
**TCRR1-5µM BA - WT-MS**	*	-1,72863	0,773302
**TCRR1-MS - TCRR2-5µM BA**		0,629205	0,84711
**TCRR1-MS - TCRR2-MS**	*	2,32161	0,889643
**TCRR1-MS - WT-5µM BA**	*	1,76823	0,773302
**TCRR1-MS - WT-MS**		0,0490855	0,773302
**TCRR2-5µM BA - TCRR2-MS**	*	1,6924	0,954497
**TCRR2-5µM BA - WT-5µM BA**	*	1,13902	0,84711
**TCRR2-5µM BA - WT-MS**		-0,580119	0,84711
**TCRR2-MS - WT-5µM BA**		-0,553379	0,889643

**TCRR2-MS - WT-MS**	*	-2,27252	0,889643
**WT-5µM BA - WT-MS**	*	-1,71914	0,773302

* denotes a statistically significant difference.

Multiple Range Tests for Primary-root length by Genotype-Treatment. Days 7+5

Method: 95,0 percent Bonferroni

Level	*Count*	*Mean*	*Homogeneous Groups*
**TCRR2-MS**	17	1,49506	X
**WT-5µM BA**	28	1,86856	X
**TCRR1-5µM BA**	28	2,0595	X
**TCRR2-5µM BA**	20	3,88886	X
**WT-MS**	28	5,65138	X
**TCRR1-MS**	28	5,66663	X

Contrast	*Sig.*	*Difference*	*+/- Limits*
**TCRR1-5µM BA - TCRR1-MS**	*	-3,60712	1,06176
**TCRR1-5µM BA - TCRR2-5µM BA**	*	-1,82935	1,1631
**TCRR1-5µM BA - TCRR2-MS**		0,564441	1,2215
**TCRR1-5µM BA - WT-5µM BA**		0,190948	1,06176
**TCRR1-5µM BA - WT-MS**	*	-3,59187	1,06176
**TCRR1-MS - TCRR2-5µM BA**	*	1,77777	1,1631
**TCRR1-MS - TCRR2-MS**	*	4,17156	1,2215
**TCRR1-MS - WT-5µM BA**	*	3,79807	1,06176
**TCRR1-MS - WT-MS**		0,0152496	1,06176
**TCRR2-5µM BA - TCRR2-MS**	*	2,3938	1,31055
**TCRR2-5µM BA - WT-5µM BA**	*	2,0203	1,1631
**TCRR2-5µM BA - WT-MS**	*	-1,76252	1,1631
**TCRR2-MS - WT-5µM BA**		-0,373493	1,2215
**TCRR2-MS - WT-MS**	*	-4,15631	1,2215
**WT-5µM BA - WT-MS**	*	-3,78282	1,06176

* denotes a statistically significant difference.
