# Supplementary material for: Distinct Hormone Signalling-Modulation Activities Characterize Two Maize Endosperm-Specific Type-A Response Regulators
Source: Plants (Basel). 2022 Jul 30;11(15):1992. doi: 10.3390/plants11151992 (PMC9370639; doi:10.3390/plants11151992)
Supplement: Supplementary file 1 [file plants-11-01992-s001.zip › Suppl Table 2.pdf]

Supplementary Table S2. Statistical analyses of the primary root length of plants grown in vertical plates in the presence or absence of NAA. For each time point statistically homogeneous groups are defined according to the pairwise comparisons using Bonferroni's test with p≤0.05.

Multiple Range Tests for Primary root by Genotype-Treatment. Days in culture: 7
Method: 95,0 percent Bonferroni

Level	*Count*	*Mean*	*Homogeneous Groups*
**Col-NAA-125**	23	1,08463	X
**Col-MS**	23	1,22678	XX
**L2-NAA-125**	23	1,23174	XX
**Col-NAA-12.5**	24	1,27665	X
**L1-NAA-125**	23	1,31582	XX
**L2-NAA-12.5**	23	1,49208	XX
**L1-NAA-12.5**	22	1,52584	X
**L1-MS**	23	1,52777	X
**L2-MS**	23	1,58822	X

Contrast	*Sig.*	*Difference*	*+/- Limits*
**Col-MS - Col-NAA-12.5**		-0,049875	0,190739
**Col-MS - Col-NAA-125**		0,142148	0,192757
**Col-MS - L1-MS**	*	-0,300996	0,192757
**Col-MS - L1-NAA-12.5**	*	-0,299065	0,194935
**Col-MS - L1-NAA-125**		-0,0890435	0,192757
**Col-MS - L2-MS**	*	-0,361439	0,192757
**Col-MS - L2-NAA-12.5**	*	-0,265299	0,192757
**Col-MS - L2-NAA-125**		-0,00496087	0,192757
**Col-NAA-12.5 - Col-NAA-125**	*	0,192023	0,190739
**Col-NAA-12.5 - L1-MS**	*	-0,251121	0,190739
**Col-NAA-12.5 - L1-NAA-12.5**	*	-0,24919	0,192939
**Col-NAA-12.5 - L1-NAA-125**		-0,0391685	0,190739
**Col-NAA-12.5 - L2-MS**	*	-0,311564	0,190739
**Col-NAA-12.5 - L2-NAA-12.5**	*	-0,215424	0,190739
**Col-NAA-12.5 - L2-NAA-125**		0,0449141	0,190739
**Col-NAA-125 - L1-MS**	*	-0,443143	0,192757
**Col-NAA-125 - L1-NAA-12.5**	*	-0,441213	0,194935
**Col-NAA-125 - L1-NAA-125**	*	-0,231191	0,192757
**Col-NAA-125 - L2-MS**	*	-0,503587	0,192757
**Col-NAA-125 - L2-NAA-12.5**	*	-0,407446	0,192757
**Col-NAA-125 - L2-NAA-125**		-0,147109	0,192757
**L1-MS - L1-NAA-12.5**		0,00193054	0,194935
**L1-MS - L1-NAA-125**	*	0,211952	0,192757
**L1-MS - L2-MS**		-0,0604435	0,192757
**L1-MS - L2-NAA-12.5**		0,0356971	0,192757
**L1-MS - L2-NAA-125**	*	0,296035	0,192757
**L1-NAA-12.5 - L1-NAA-125**	*	0,210022	0,194935
**L1-NAA-12.5 - L2-MS**		-0,062374	0,194935
**L1-NAA-12.5 - L2-NAA-12.5**		0,0337666	0,194935
**L1-NAA-12.5 - L2-NAA-125**	*	0,294104	0,194935
**L1-NAA-125 - L2-MS**	*	-0,272396	0,192757
**L1-NAA-125 - L2-NAA-12.5**		-0,176255	0,192757
**L1-NAA-125 - L2-NAA-125**		0,0840826	0,192757
**L2-MS - L2-NAA-12.5**		0,0961406	0,192757
**L2-MS - L2-NAA-125**	*	0,356478	0,192757
**L2-NAA-12.5 - L2-NAA-125**	*	0,260338	0,192757

* denotes a statistically significant difference.

Multiple Range Tests for Primary root by Genotype-Treatment. Days in culture: 10

Method: 95,0 percent Bonferroni

Level	*Count*	*Mean*	*Homogeneous Groups*
**Col-NAA-125**	23	2,72778	X
**L2-NAA-125**	23	3,00709	XX
**L1-NAA-125**	23	3,11052	X
**Col-NAA-12.5**	24	3,19471	X
**Col-MS**	23	3,29033	X
**L2-NAA-12.5**	22	3,66291	X
**L1-NAA-12.5**	20	3,67112	X
**L1-MS**	21	3,93608	XX
**L2-MS**	23	4,05683	X

Contrast	*Sig.*	*Difference*	*+/- Limits*
**Col-MS - Col-NAA-12.5**		0,0956203	0,29756
**Col-MS - Col-NAA-125**	*	0,562552	0,300709
**Col-MS - L1-MS**	*	-0,645741	0,307786
**Col-MS - L1-NAA-12.5**	*	-0,380784	0,311782
**Col-MS - L1-NAA-125**		0,179813	0,300709
**Col-MS - L2-MS**	*	-0,7665	0,300709
**Col-MS - L2-NAA-12.5**	*	-0,372577	0,304107
**Col-MS - L2-NAA-125**		0,283243	0,300709
**Col-NAA-12.5 - Col-NAA-125**	*	0,466932	0,29756
**Col-NAA-12.5 - L1-MS**	*	-0,741362	0,30471
**Col-NAA-12.5 - L1-NAA-12.5**	*	-0,476404	0,308746
**Col-NAA-12.5 - L1-NAA-125**		0,0841928	0,29756
**Col-NAA-12.5 - L2-MS**	*	-0,86212	0,29756
**Col-NAA-12.5 - L2-NAA-12.5**	*	-0,468198	0,300994
**Col-NAA-12.5 - L2-NAA-125**		0,187623	0,29756
**Col-NAA-125 - L1-MS**	*	-1,20829	0,307786
**Col-NAA-125 - L1-NAA-12.5**	*	-0,943336	0,311782
**Col-NAA-125 - L1-NAA-125**	*	-0,382739	0,300709
**Col-NAA-125 - L2-MS**	*	-1,32905	0,300709
**Col-NAA-125 - L2-NAA-12.5**	*	-0,93513	0,304107
**Col-NAA-125 - L2-NAA-125**		-0,279309	0,300709
**L1-MS - L1-NAA-12.5**		0,264957	0,318613
**L1-MS - L1-NAA-125**	*	0,825554	0,307786
**L1-MS - L2-MS**		-0,120759	0,307786
**L1-MS - L2-NAA-12.5**		0,273164	0,311107
**L1-MS - L2-NAA-125**	*	0,928985	0,307786
**L1-NAA-12.5 - L1-NAA-125**	*	0,560597	0,311782
**L1-NAA-12.5 - L2-MS**	*	-0,385716	0,311782
**L1-NAA-12.5 - L2-NAA-12.5**		0,00820667	0,315061
**L1-NAA-12.5 - L2-NAA-125**	*	0,664028	0,311782
**L1-NAA-125 - L2-MS**	*	-0,946313	0,300709
**L1-NAA-125 - L2-NAA-12.5**	*	-0,55239	0,304107
**L1-NAA-125 - L2-NAA-125**		0,10343	0,300709
**L2-MS - L2-NAA-12.5**	*	0,393923	0,304107
**L2-MS - L2-NAA-125**	*	1,04974	0,300709
**L2-NAA-12.5 - L2-NAA-125**	*	0,655821	0,304107

* denotes a statistically significant difference.

Multiple Range Tests for Primary root by Genotype-Treatment. Days in culture: 13

Method: 95,0 percent Bonferroni

Level	*Count*	*Mean*	*Homogeneous Groups*
**Col-NAA-125**	23	5,11033	X
**L2-NAA-125**	23	5,43938	XX
**L1-NAA-125**	23	5,60527	X
**Col-NAA-12.5**	24	6,09296	X
**Col-MS**	23	6,16083	X
**L1-NAA-12.5**	20	6,89276	X
**L2-NAA-12.5**	22	6,93421	XX
**L1-MS**	21	7,26068	XX
**L2-MS**	23	7,33977	X

Contrast	*Sig.*	*Difference*	*+/- Limits*
**Col-MS - Col-NAA-12.5**		0,0678611	0,426132
**Col-MS - Col-NAA-125**	*	1,0505	0,430641
**Col-MS - L1-MS**	*	-1,09985	0,440776
**Col-MS - L1-NAA-12.5**	*	-0,731934	0,446499
**Col-MS - L1-NAA-125**	*	0,555557	0,430641
**Col-MS - L2-MS**	*	-1,17894	0,430641
**Col-MS - L2-NAA-12.5**	*	-0,773387	0,435508
**Col-MS - L2-NAA-125**	*	0,721448	0,430641
**Col-NAA-12.5 - Col-NAA-125**	*	0,982639	0,426132
**Col-NAA-12.5 - L1-MS**	*	-1,16772	0,436371
**Col-NAA-12.5 - L1-NAA-12.5**	*	-0,799796	0,442151
**Col-NAA-12.5 - L1-NAA-125**	*	0,487695	0,426132
**Col-NAA-12.5 - L2-MS**	*	-1,2468	0,426132
**Col-NAA-12.5 - L2-NAA-12.5**	*	-0,841248	0,431049
**Col-NAA-12.5 - L2-NAA-125**	*	0,653587	0,426132
**Col-NAA-125 - L1-MS**	*	-2,15035	0,440776
**Col-NAA-125 - L1-NAA-12.5**	*	-1,78243	0,446499
**Col-NAA-125 - L1-NAA-125**	*	-0,494943	0,430641
**Col-NAA-125 - L2-MS**	*	-2,22944	0,430641
**Col-NAA-125 - L2-NAA-12.5**	*	-1,82389	0,435508
**Col-NAA-125 - L2-NAA-125**		-0,329052	0,430641
**L1-MS - L1-NAA-12.5**		0,36792	0,456281
**L1-MS - L1-NAA-125**	*	1,65541	0,440776
**L1-MS - L2-MS**		-0,0790843	0,440776
**L1-MS - L2-NAA-12.5**		0,326468	0,445531
**L1-MS - L2-NAA-125**	*	1,8213	0,440776
**L1-NAA-12.5 - L1-NAA-125**	*	1,28749	0,446499
**L1-NAA-12.5 - L2-MS**	*	-0,447005	0,446499
**L1-NAA-12.5 - L2-NAA-12.5**		-0,0414523	0,451194
**L1-NAA-12.5 - L2-NAA-125**	*	1,45338	0,446499
**L1-NAA-125 - L2-MS**	*	-1,7345	0,430641
**L1-NAA-125 - L2-NAA-12.5**	*	-1,32894	0,435508
**L1-NAA-125 - L2-NAA-125**		0,165891	0,430641
**L2-MS - L2-NAA-12.5**		0,405552	0,435508
**L2-MS - L2-NAA-125**	*	1,90039	0,430641
**L2-NAA-12.5 - L2-NAA-125**	*	1,49483	0,435508

* denotes a statistically significant difference.
