# Supplementary material for: Distinct Hormone Signalling-Modulation Activities Characterize Two Maize Endosperm-Specific Type-A Response Regulators
Source: Plants (Basel). 2022 Jul 30;11(15):1992. doi: 10.3390/plants11151992 (PMC9370639; doi:10.3390/plants11151992)
Supplement: Supplementary file 1 [file plants-11-01992-s001.zip › Suppl Table 3.pdf]

PRIMER ID	SEQUENCE 5'-3'	PURPOSE, COMMENTS
**Gateway\attB1**	GGGGACAAGTTTGTACAAAAAAGCAGGCT	Invitrogen custom Gateway PCR Primer
**Gateway\attB2**	GGGGACCACTTTGTACAAGAAAGCTGGGT	Invitrogen custom Gateway PCR Primer
**RRLp-GWAS**	AGAAAGCTGGGTAGATACTCTCCCACAACTTCC	Cloning of zmTCRR2 promoter
**RRLp-GWS**	AAAAAGCAGGCTTAGTGTGCAATCGAAGCAACGG	Cloning of zmTCRR2 promoter
**RRQPCR-AS3**	CGATTCCTTCACTTCCCTAA	qRTPCR quantitation of ZmTCRR1
**RRQPCR-S3**	GCATTGGAATTCTTAGATGCGAAC	qRTPCR quantitation of ZmTCRR1
**QPCRActin28-S3**	CCTCAGCACTTTCCAGCAGATGTG	Normalizer for the quantitation of ZmTCRR1
**QPCRActin28-AS3**	TTTCTGTGGACAATGCCTGGACC	Normalizer for the quantitation of ZmTCRR1

Supplementary Table S3. Oligonucleotides used in this study.
